# Supplementary material for: Copaifera langsdorffii Oleoresin-Loaded Nanostructured Lipid Carrier Emulgel Improves Cutaneous Healing by Anti-Inflammatory and Re-Epithelialization Mechanisms
Source: Int J Mol Sci. 2023 Nov 1;24(21):15882. doi: 10.3390/ijms242115882 (PMC10648863; doi:10.3390/ijms242115882)
Supplement: Supplementary file 1 [file ijms-24-15882-s001.zip › ijms-2610112-supplementary.pdf]

Supplementary materials

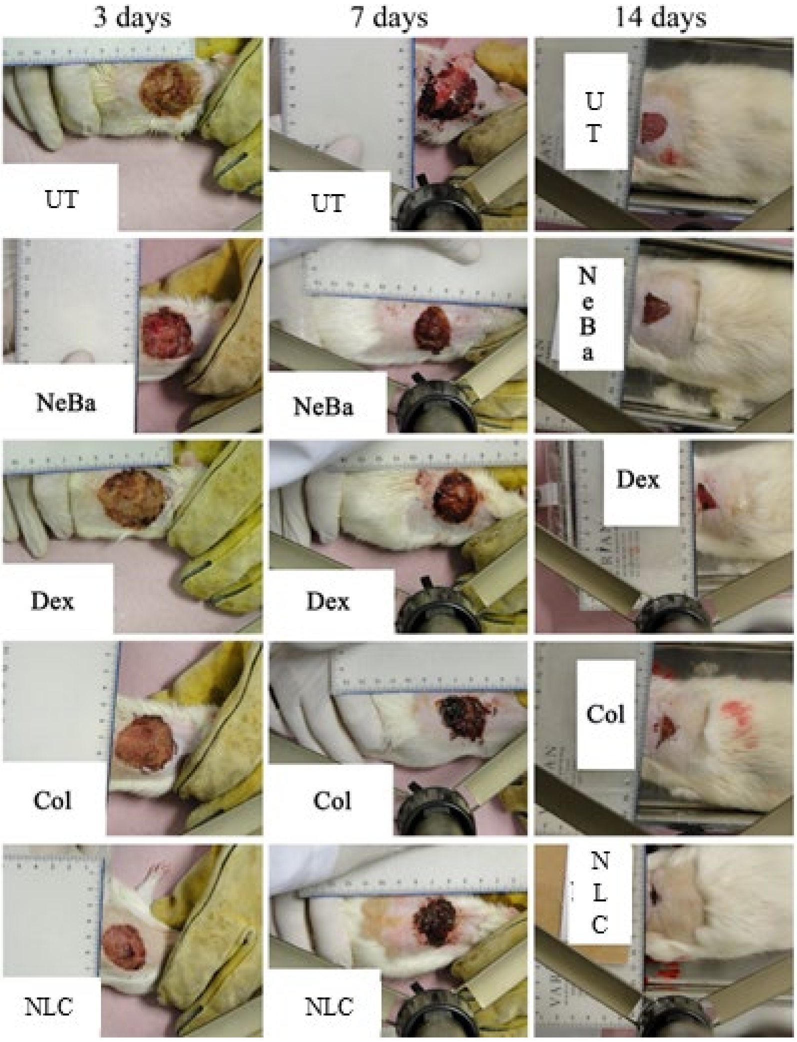

FIGURE S1: Photographs of skin wounds of the groups UT, NeBa, Dex, Col and NLC during 3, 7 and 14 days of experimentation. UT: wounded animals without treatment. NeBa: wounded animals treated with neomycin + sulfate bacitracin zinc. Dex: wounded animals treated with dexpanthenol. Col: wounded animals treated with collagenase. NLC: wounded animals treated with 1% *Copaifera langsdorffii* oleoresin loaded in nanostructured lipid carriers.

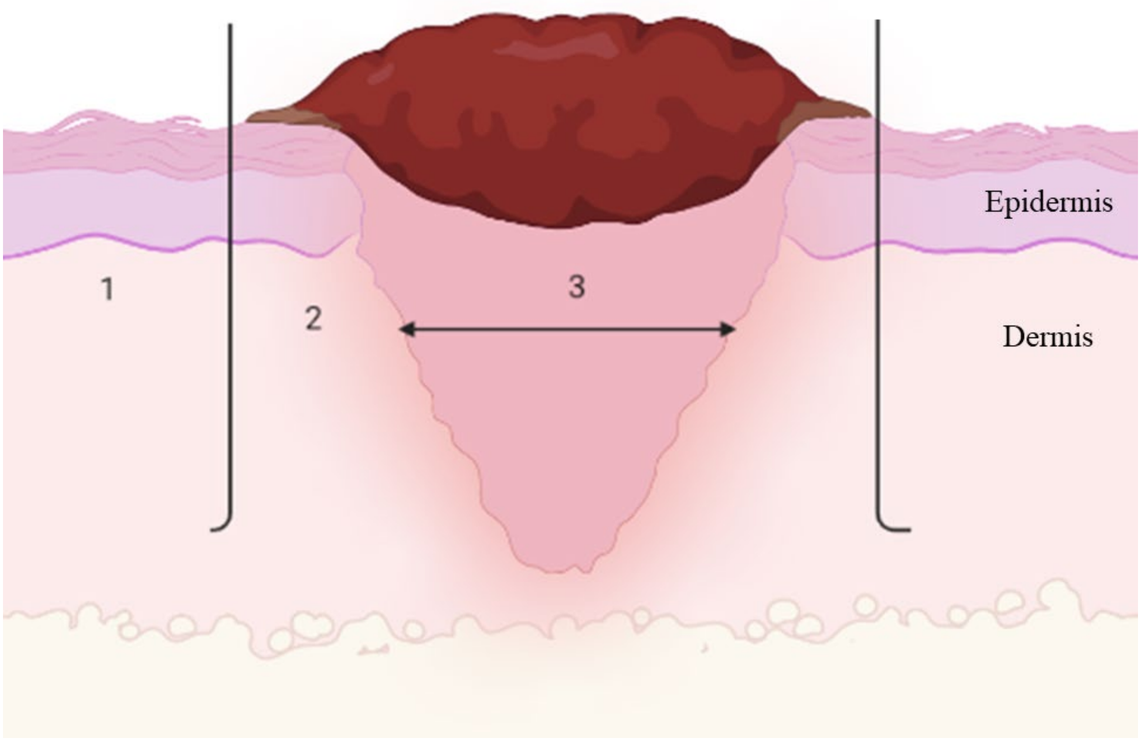

FIGURE S2: Representative scheme of cutaneous wound demonstrating the three regions of the microscopic analysis: (1) normal skin - without lesion, (2) border and (3) center of the wound.
